# Trends in Violence Victimization and Suicide Risk by Sexual Identity Among High School Students — Youth Risk Behavior Survey, United States, 2015–2019

**DOI:** 10.15585/mmwr.su6901a3

**Published:** 2020-08-21

**Authors:** Michelle M. Johns, Richard Lowry, Laura T. Haderxhanaj, Catherine N. Rasberry, Leah Robin, Lamont Scales, Deborah Stone, Nicolas A. Suarez

**Affiliations:** ^1^Division of Adolescent and School Health, National Center for HIV/AIDS, Viral Hepatitis, STD, and TB Prevention, CDC; ^2^Office of the Director, National Center for HIV/AIDS, Viral Hepatitis, STD, and TB Prevention, CDC; ^3^Division of STD Prevention, National Center for HIV/AIDS, Viral Hepatitis, STD, and TB Prevention, CDC; ^4^Division of HIV/AIDS Prevention, National Center for HIV/AIDS, Viral Hepatitis, STD, and TB Prevention, CDC; ^5^Division of Injury Prevention, National Center for Injury Prevention and Control, CDC

## Abstract

Lesbian, gay, and bisexual (LGB) youths continue to experience more violence victimization and suicide risk than heterosexual youths; however, few studies have examined whether the proportion of LGB youths affected by these outcomes has varied over time, and no studies have assessed such trends in a nationally representative sample. This report analyzes national trends in violence victimization and suicide risk among high school students by self-reported sexual identity (LGB or heterosexual) and evaluates differences in these trends among LGB students by sex (male or female) and race/ethnicity (non-Hispanic black, non-Hispanic white, or Hispanic). Data for this analysis were derived from the 2015, 2017, and 2019 cycles of CDC’s Youth Risk Behavior Survey (YRBS), a cross-sectional, school-based survey conducted biennially since 1991. Logistic regression models assessed linear trends in prevalence of violence victimization and indicators of suicide risk among LGB and heterosexual students during 2015–2019; in subsequent models, sex-stratified (controlling for race/ethnicity and grade) and race/ethnicity-stratified (controlling for sex and grade) linear trends were examined for students self-identifying as LGB during 2015–2019. Results demonstrated that LGB students experienced more violence victimization and reported more suicide risk behaviors than heterosexual youths. Among LGB youths, differences in the proportion reporting violence victimization and suicide risk by sex and race/ethnicity were found. Across analyses, very few linear trends in these outcomes were observed among LGB students. Results highlight the continued need for comprehensive intervention strategies within schools and communities with the express goal of reducing violence victimization and preventing suicide risk behaviors among LGB students.

## Introduction

Lesbian, gay, and bisexual (LGB) youths experience more violence victimization and suicide risk than heterosexual youths (*1*–*3*). In 2015, CDC’s Youth Risk Behavior Surveillance System (YRBSS) added two new questions to the national questionnaire regarding sexual identity and sexual behavior. These questions facilitated the first nationally representative estimates of the health behaviors and experiences of sexual minority youths (students who identify as LGB or those who have sexual contact with persons of the same or both sexes) and affirmed the presence of substantial health disparities (i.e., differences in health outcomes between social groups driven by unequal social or environmental circumstances) in violence victimization and suicide risk between LGB and heterosexual youths . Findings from the 2017 Youth Risk Behavior Survey (YRBS) revealed that LGB high school students experienced more bullying at school (33% among LGB students and 17% among heterosexual students), more sexual dating violence by dating partners (LGB, 16%; heterosexual, 6%), and more suicide attempts (LGB, 23%; heterosexual, 5%) (*3*) than their heterosexual peers.

Notably, the 2019 YRBS data collection cycle presented the first opportunity for examining linear trends in violence victimization and suicide risk trends for LGB students across time in a nationally representative sample. Few studies have examined whether prevalence of violence victimization and suicide risk varies among LGB youths over time (*4*). School environments in the United States might be improving in their ability to meet the needs of LGB youths (*5*); for example, recent surveillance data from CDC’s 2018 School Health Profiles, which include representative data from 43 states, provide evidence that many schools are implementing supportive practices. In the 2018 School Health Profiles, an average of 78.5% of schools across U.S. states included in the sample reported identifying safe spaces for LGB and transgender and questioning youths, and an average of 96.1% of schools across these states prohibited harassment based on a student’s perceived or actual sexual orientation or gender identity (*6*). A recent study (*4*) that pooled local YRBS data during 2009–2017 to examine trends in suicide risk found that reports among LGB youths of suicide risk might be decreasing but that LGB students still are as much as three times more likely to have attempted suicide than heterosexual students. National U.S. trends have not been estimated.

LGB youths are a heterogenous population with intersecting social identities (e.g., sex, race/ethnicity, or gender identity), and important differences might exist among LGB youths regarding risk for violence and suicide. For example, LGB females appear to be at higher risk for dating and sexual violence than LGB males (*7*,*8*). In examinations of racial/ethnic differences among LGB youths, clear patterns of differences in experiences of violence victimization and suicide risk are less consistent (*9*,*10*). For example, one study of interpersonal violence among sexual minorities reported that physical victimization from an intimate partner was 1–4 times higher among non-white youths than among white youths (*9*). Another study reported that non-Hispanic white and Hispanic LGB youths were more likely to be bullied than non-Hispanic white heterosexual youths but that non-Hispanic black LGB youths were not more likely to be bullied than non-Hispanic white heterosexual youths (*10*). This same study reported that all LGB youths, regardless of race/ethnicity, were at increased risk for suicidal ideation (*10*). More systematic evaluations of the within-group differences in violence victimization and suicide risk behaviors among sexual minority youths are warranted.

This analysis contributes to the evidence base regarding LGB students, violence victimization, and suicide risk. YRBS data were used to examine national trends in violence victimization and suicide risk among high school students by self-reported sexual identity and evaluated differences among LGB students by sex and race/ethnicity. The analysis was guided by the following four questions:

1. How did the prevalence of violence victimization and suicide risk among LGB students vary during 2015–2019?
2. To what extent did violence victimization and suicide risk trends differ from these trends among heterosexual students during the same period?
3. Among LGB students, to what extent did violence victimization and suicide risk trends vary by sex (male or female)?
4. Among LGB students, to what extent did violence victimization and suicide risk trends vary by race/ethnicity (non-Hispanic black, non-Hispanic white, or Hispanic)?

## Methods

### Data Source

This report includes data from the 2015 (n = 15,624), 2017 (n = 14,765), and 2019 (n = 13,677) cycles of the national YRBS (pooled n = 44,066), a cross-sectional, school-based survey conducted biennially since 1991. Each survey year, CDC collects data from a nationally representative sample of public and private school students in grades 9–12 in the 50 U.S. states and the District of Columbia. Additional information about YRBS sampling, data collection, response rates, and processing is available in the overview report of this supplement (*11*). The prevalence estimates for all questions on violence victimization and suicide risk for the overall study population and by sex, race/ethnicity, grade, and sexual orientation are available at https://nccd.cdc.gov/youthonline/App/Default.aspx. The full YRBS questionnaire is available at https://www.cdc.gov/healthyyouth/data/yrbs/pdf/2019/2019_YRBS-National-HS-Questionnaire.pdf.

### Measures

All measures analyzed for this report are provided (Table 1). Students responded to seven questions about violence victimization, including ever experiencing forced sexual intercourse; experiencing sexual dating violence, physical dating violence, bullying at school, electronic bullying, and being threatened or injured with a weapon at school during the previous 12 months; and missing school because of feeling unsafe at or on the way to or from school during the previous 30 days. Students responded to five questions about suicide risk during the previous 12 months, including having felt persistently sad or hopeless; having seriously considered suicide; and having made a suicide plan, having attempted suicide, or having made a suicide attempt that had to be treated by a doctor or nurse. Students responded to five demographic questions relating to sex, sexual identity, grade, race, and ethnicity, which were used as covariates and to create relevant strata in all trend analyses.

**TABLE 1:** Measures for demographic characteristics, violence victimization, and suicide risk behaviors among high school students — Youth Risk Behavior Survey, United States, 2019

Construct	Measure
**Demographic characteristics**	
Sexual identity	Which of the following best describes you? A. Heterosexual (straight) B. Gay or lesbian C. Bisexual D. Not sure
Sex at birth	What is your sex? A. Female B. Male
Race	What is your race? (*Select one or more responses.*) A. American Indian or Alaska Native B. Asian C. Black or African American D. Native Hawaiian or Other Pacific Islander E. White
Ethnicity	Are you Hispanic or Latino? A. Yes B. No
Grade	In what grade are you? A. 9th grade B. 10th grade C. 11th grade D. 12th grade E. Ungraded or other grade
Violence victimization*	
Forced sex	Have you ever been physically forced to have sexual intercourse when you did not want to?
Sexual dating violence	During the past 12 months, how many times did someone you were dating or going out with force you to do sexual things that you did not want to do? (Count such things as kissing, touching, or being physically forced to have sexual intercourse.)
Physical dating violence	During the past 12 months, how many times did someone you were dating or going out with physically hurt you on purpose? (Count such things as being hit, slammed into something, or injured with an object or weapon.)
Bullying at school	During the past 12 months, have you ever been bullied on school property?
Electronic bullying	During the past 12 months, have you ever been electronically bullied? (Count being bullied through texting, Instagram, Facebook, or other social media.)
Felt unsafe at, to, or from school	During the past 30 days, on how many days did you not go to school because you felt you would be unsafe at school or on your way to or from school?
Threatened or injured with a weapon at school	During the past 12 months, how many times has someone threatened or injured you with a weapon, such as a gun, knife, or club, on school property?
Suicide risk behaviors*	
Persistent feelings of sadness/hopelessness	During the past 12 months, did you ever feel so sad or hopeless almost every day for 2 weeks or more in a row that you stopped doing some usual activities?
Seriously considered suicide	During the past 12 months, did you ever seriously consider attempting suicide?
Made a suicide plan	During the past 12 months, did you make a plan about how you would attempt suicide?
Attempted suicide	During the past 12 months, how many times did you actually attempt suicide?
Suicide attempt requiring medical treatment	If you attempted suicide during the past 12 months, did any attempt result in an injury, poisoning, or overdose that had to be treated by a doctor or nurse?

* All violence victimization and suicide risk measures were dichotomized as either “yes” (i.e., ≥1 time, ≥1 day) or “no” (i.e., 0 days, 0 times).

### Analysis

Data from the 2015, 2017, and 2019 national YRBS were examined for trends in the prevalence among LGB students in experiences of violence victimization and indicators of suicide risk. Data were analyzed by using SAS (version 9.4; SAS Institute) and SUDAAN (version 11.0.0; RTI International) to account for the complex sampling designs. Data were assessed using complete case analysis; missing data were not imputed. All outcomes were dichotomized as either yes or no, ≥1 time or 0 times, or ≥1 day or 0 days. Weighted prevalence estimates with 95% confidence intervals (CIs) were calculated by using Taylor series linearization to produce nationally representative prevalence estimates for each survey year.

Logistic regression models were used to assess linear trends in the prevalence of violence victimization and indicators of suicide risk among LGB and heterosexual students for 2015–2019, controlling for sex, race/ethnicity, and grade. Main effects odds ratios (ORs) comparing LGB students with heterosexual students also were calculated for the 2015–2019 period. In subsequent models, sex-stratified (controlling for race/ethnicity and grade) and race/ethnicity-stratified (controlling for sex and grade) linear trends, were examined for students self-identifying as LGB on the survey. Main effects ORs comparing sex and race/ethnicity groups also were calculated for these subsequent regression models. Linear trends were considered statistically significant if p<0.05. Main effects ORs were considered statistically significant if 95% CIs did not include 1.0.

## Results

### Violence Victimization

Among all students (Table 2), LGB students had greater odds of violence victimization than heterosexual students across all seven indicators, as evidenced by statistically significant main effects of sexual identity on each indicator (Table 2). Among LGB students, the percentage who reported experiencing physical dating violence during 2015–2019 significantly decreased from 17.5% to 13.1%. No other violence victimization outcomes varied significantly among LGB students in this period.

**TABLE 2:** Trends in the prevalence of violence victimization and suicide risk behaviors among high school students, by self-identified sexual identity — Youth Risk Behavior Survey, United States, 2015–2019*

Health risk behavior	Main effect	2015	2017	2019	Linear trend	
	aOR (95% CI)	% (95% CI)	% (95% CI)	% (95% CI)	Beta	p value^†^
**Violence victimization**						
Feeling unsafe at school (past 30 days)						
Lesbian, gay, or bisexual	1.98 (1.70–2.30)	12.5 (10.2–15.3)	10.0 (8.1–12.3)	13.5 (11.0–16.5)	0.0619	0.60
Heterosexual	1.0 (Ref.)	4.6 (3.9–5.4)	6.1 (5.1–7.3)	7.5 (6.3–8.9)	0.3749	0.00
Threatened or injured with a weapon at school (past 12 months)						
Lesbian, gay, or bisexual	2.09 (1.80–2.43)	10.0 (7.9–12.7)	9.4 (7.4–11.8)	11.9 (9.3–15.2)	0.2463	0.12
Heterosexual	1.0 (Ref.)	5.1 (4.5–5.9)	5.4 (4.8–6.0)	6.3 (5.5–7.3)	0.1629	0.02
Bullied at school (past 12 months)						
Lesbian, gay, or bisexual	2.10 (1.87–2.37)	34.2 (29.6–39.0)	33.0 (27.4–39.0)	32.0 (29.5–34.6)	–0.0847	0.28
Heterosexual	1.0 (Ref.)	18.8 (17.3–20.3)	17.1 (16.1–18.2)	17.1 (15.7–18.7)	–0.0800	0.10
Electronically bullied (past 12 months)						
Lesbian, gay, or bisexual	1.94 (1.72–2.20)	28.0 (24.0–32.3)	27.1 (23.1–31.4)	26.6 (23.3–30.2)	–0.0775	0.43
Heterosexual	1.0 (Ref.)	14.2 (13.1–15.3)	13.3 (12.4–14.4)	14.1 (12.9–15.4)	–0.0047	0.92
Physical dating violence (past 12 months)						
Lesbian, gay, or bisexual	2.06 (1.77–2.40)	17.5 (14.4–21.2)	17.2 (14.3–20.5)	13.1 (10.5–16.1)	–0.2264	0.04
Heterosexual	1.0 (Ref.)	8.3 (7.5–9.3)	6.4 (5.8–7.1)	7.2 (6.2–8.3)	–0.1448	0.047
Sexual dating violence (past 12 months)						
Lesbian, gay, or bisexual	2.08 (1.69–2.57)	22.7 (18.0–28.2)	15.8 (12.3–20.1)	16.4 (12.7–20.9)	–0.2420	0.15
Heterosexual	1.0 (Ref.)	9.1 (8.2–10.0)	5.5 (4.8–6.3)	6.7 (5.9–7.5)	–0.2785	<0.001
Forced sexual intercourse (lifetime)						
Lesbian, gay, or bisexual	3.31 (2.90–3.77)	17.8 (14.4–21.8)	21.9 (19.0–25.0)	19.4 (16.2–23.1)	0.0650	0.59
Heterosexual	1.0 (Ref.)	5.4 (4.6–6.4)	5.4 (4.7–6.2)	5.5 (4.9–6.2)	0.0200	0.82
**Suicide risk behaviors**						
Persistent feelings of sadness or hopelessness (past 12 months)						
Lesbian, gay, or bisexual	3.60 (3.22–4.03)	60.4 (55.1–65.4)	63.0 (59.5–66.5)	66.3 (62.2–70.2)	0.1566	0.13
Heterosexual	1.0 (Ref.)	26.4 (24.6–28.4)	27.5 (25.9–29.2)	32.2 (30.8–33.7)	0.1949	0.00
Seriously considered attempting suicide (past 12 months)						
Lesbian, gay, or bisexual	4.51 (4.07–4.99)	42.8 (38.4–47.3)	47.7 (43.7–51.8)	46.8 (43.1–50.6)	0.0936	0.26
Heterosexual	1.0 (Ref.)	14.8 (13.7–15.9)	13.3 (12.5–14.3)	14.5 (13.4–15.7)	–0.0242	0.62
Made a suicide plan (past 12 months)						
Lesbian, gay, or bisexual	4.28 (3.84–4.77)	38.2 (34.0–42.6)	38.0 (34.5–41.7)	40.2 (36.6–44.0)	0.0646	0.45
Heterosexual	1.0 (Ref.)	11.9 (10.8–13.1)	10.4 (9.3–11.7)	12.1 (11.1–13.1)	0.0031	0.96
Attempted suicide (past 12 months)						
Lesbian, gay, or bisexual	4.54 (3.89–5.28)	29.4 (25.7–33.3)	23.0 (18.6–28.0)	23.4 (20.0–27.1)	–0.1901	0.06
Heterosexual	1.0 (Ref.)	6.4 (5.6–7.3)	5.4 (4.6–6.4)	6.4 (5.6–7.4)	–0.0148	0.85
Suicide attempt requiring medical treatment (past 12 months)						
Lesbian, gay, or bisexual	3.78 (3.02–4.73)	9.4 (7.3–12.1)	7.5 (5.7–9.8)	6.3 (4.8–8.3)	–0.2852	0.07
Heterosexual	1.0 (Ref.)	2.0 (1.5–2.7)	1.7 (1.4–2.1)	1.7 (1.4–2.2)	–0.1197	0.40

**Abbreviations:** aOR = adjusted odds ratio; CI = confidence interval; Ref. = referent group.

* Logistic regression models were used to assess linear trends in the prevalence of violence victimization, and indicators of suicide risk among lesbian, gay, or bisexual students and heterosexual students for 2015–2019, controlling for sex, race/ethnicity, and grade.

^†^ Statistical significance is defined as p<0.05 or a 95% CI that does not include 1.0.

Among LGB students stratified by sex (Table 3), male students reported greater odds of feeling unsafe at or on the way to or from school (aOR: 1.61) and being threatened or injured with a weapon (aOR: 1.54) than female students. Conversely, male LGB students reported reduced odds of electronic bullying (aOR: 0.71), sexual dating violence (aOR: 0.66), and forced sex (aOR: 0.51) than female LGB students. Among male LGB students, the percentage reporting being threatened or injured with a weapon at school significantly increased from 2015 (11.6%) to 2019 (15.9%), as did the percentage reporting forced sex (2015: 8.0%; 2019: 15.6%). Among female LGB students, the percentage reporting physical dating violence significantly decreased from 2015 (16.9%) to 2019 (12.1%).

**TABLE 3:** Trends in violence victimization and suicide risk behaviors among lesbian, gay, and bisexual high school students, by sex and sexual identity — Youth Risk Behavior Survey, United States, 2015–2019*

Health risk behavior	Main effect	2015	2017	2019	Linear trend	
	aOR (95% CI)	% (95% CI)	% (95% CI)	% (95% CI)	Beta	p value^†^
**Violence victimization**						
Feeling unsafe at school (past 30 days)						
Gay or bisexual male	1.61 (1.14–2.28)	15.5 (9.5–24.4)	12.3 (7.4–19.6)	18.3 (12.4–26.1)	0.1623	0.55
Lesbian or bisexual female	1.0 (Ref.)	10.8 (8.6–13.5)	9.1 (6.9–11.9)	11.5 (9.5–14.0)	0.0435	0.72
Threatened or injured with a weapon at school (past 12 months)						
Gay or bisexual male	1.54 (1.14–2.08)	11.6 (7.5–17.5)	14.6 (9.8–21.2)	15.9 (11.4–21.8)	0.3973	0.04
Lesbian or bisexual female	1.0 (Ref.)	9.1 (6.6–12.4)	7.4 (5.6–9.7)	10.6 (8.1–13.9)	0.1944	0.29
Bullied at school (past 12 months)						
Gay or bisexual male	0.86 (0.69–1.08)	26.3 (19.4–34.7)	35.0 (25.4–45.9)	31.7 (25.7–38.4)	0.0942	0.58
Lesbian or bisexual female	1.0 (Ref.)	37.2 (32.7–42.0)	32.2 (26.9–38.1)	32.0 (28.6–35.7)	–0.1550	0.09
Electronically bullied (past 12 months)						
Gay or bisexual male	0.71 (0.57–0.89)	22.4 (16.3–30.1)	22.3 (16.5–29.4)	25.5 (18.7–33.8)	0.0786	0.71
Lesbian or bisexual female	1.0 (Ref.)	30.5 (26.0–35.4)	28.5 (24.4–33.1)	27.1 (23.7–30.7)	–0.1076	0.28
Physical dating violence (past 12 months)						
Gay or bisexual male	1.06 (0.72–1.58)	19.9 (12.9–29.4)	16.8 (10.0–27.0)	15.9 (9.4–25.6)	–0.0798	0.78
Lesbian or bisexual female	1.0 (Ref.)	16.9 (13.9–20.4)	16.9 (13.5–21.0)	12.1 (9.3–15.6)	–0.2638	0.04
Sexual dating violence (past 12 months)						
Gay or bisexual male	0.66 (0.44–0.98)	20.9 (12.7–32.6)	13.5 (7.5–23.0)	10.3 (5.6–18.3)	–0.4638	0.20
Lesbian or bisexual female	1.0 (Ref.)	22.6 (18.0–27.9)	16.3 (12.8–20.6)	18.2 (13.6–23.8)	–0.2166	0.18
Forced sexual intercourse (lifetime)						
Gay or bisexual male	0.51 (0.38–0.68)	8.0 (4.8–13.1)	15.6 (10.3–22.9)	15.6 (10.7–22.0)	0.4388	0.047
Lesbian or bisexual female	1.0 (Ref.)	21.1 (17.0–25.9)	23.7 (20.6–27.2)	21.0 (17.3–25.4)	–0.0203	0.87
**Suicide risk behaviors**						
Persistent feelings of sadness or hopelessness (past 12 months)						
Gay or bisexual male	0.39 (0.33–0.47)	43.9 (35.9–52.3)	45.5 (38.9–52.2)	53.5 (46.3–60.4)	0.2667	0.12
Lesbian or bisexual female	1.0 (Ref.)	66.5 (61.4–71.2)	68.8 (65.1–72.2)	70.5 (66.6–74.2)	0.1167	0.25
Seriously considered attempting suicide (past 12 months)						
Gay or bisexual male	0.59 (0.47–0.73)	32.7 (23.6–43.3)	37.0 (31.5–42.8)	40.4 (33.9–47.2)	0.1960	0.30
Lesbian or bisexual female	1.0 (Ref.)	46.6 (42.1–51.1)	51.0 (46.1–55.9)	49.0 (44.8–53.3)	0.0553	0.55
Made a suicide plan (past 12 months)						
Gay or bisexual male	0.57 (0.46–0.71)	27.0 (20.3–34.9)	28.7 (22.8–35.5)	33.0 (26.4–40.3)	0.2350	0.21
Lesbian or bisexual female	1.0 (Ref.)	42.0 (37.1–47.2)	40.8 (36.8–45.0)	42.4 (38.4–46.4)	0.0130	0.89
Attempted suicide (past 12 months)						
Gay or bisexual male	0.73 (0.55–0.96)	19.4 (13.6–27.0)	18.3 (11.5–27.9)	23.8 (17.8–31.1)	0.1626	0.45
Lesbian or bisexual female	1.0 (Ref.)	32.8 (28.1–37.9)	23.7 (19.4–28.5)	23.6 (20.0–27.6)	–0.2929	0.01
Suicide attempt requiring medical treatment (past 12 months)						
Gay or bisexual male	0.63 (0.42–0.97)	7.0 (3.6–13.1)	3.8 (1.9–7.3)	5.9 (3.2–10.6)	–0.2535	0.53
Lesbian or bisexual female	1.0 (Ref.)	10.3 (7.8–13.4)	8.2 (6.2–10.7)	6.6 (5.0–8.7)	–0.2977	0.05

**Abbreviations:** aOR = adjusted odds ratio; CI = confidence interval; Ref. = referent group.

^*^ Logistic regression models were used to assess linear trends in the prevalence of violence victimization and indicators of suicide risk among lesbian, gay, and bisexual high school students, by sex and self-identified sexual identity for 2015–2019, controlling for race/ethnicity and grade.

^†^ Statistical significance is defined as p<0.05 or a 95% CI that does not include 1.0.

Among LGB students stratified by race (Table 4), non-Hispanic black (black) and Hispanic students reported higher odds of feeling unsafe at or on the way to or from school than non-Hispanic white (white) students (aOR: 1.63 and aOR: 1.46, respectively), and black students also reported greater odds of being threatened or injured with a weapon than white students (aOR: 1.60). With regard to bullying, black and Hispanic LGB students reported reduced odds of both bullying at school (black, aOR: 0.31; Hispanic, aOR: 0.56) and electronic bullying (black, aOR: 0.41; Hispanic, aOR: 0.55), compared with white LGB students. Black LGB students also reported reduced odds of sexual dating violence, compared with white LGB students (aOR: 0.44). The only significant trend among violence models stratified by race/ethnicity was among Hispanic LGB students, who had reduced percentage of reporting experiencing physical dating violence in 2019 (9.8%), compared with 2015 (22.6%).

**TABLE 4:** Trends in violence victimization and suicide risk behaviors among lesbian, gay, and bisexual high school students, by race/ethnicity — Youth Risk Behavior Survey, United States, 2015–2019*

Health risk behavior	Main effect	2015	2017	2019	Linear trend	
	aOR (95% CI)	% (95% CI)	% (95% CI)	% (95% CI)	Beta	p value^†^
**Violence victimization**						
Feeling unsafe at school (past 30 days)						
Black, non-Hispanic	1.63 (1.13–2.35)	17.8 (11.4–26.6)	12.5 (7.4–20.4)	15.2 (8.3–26.2)	–0.0269	0.93
Hispanic	1.46 (1.07–1.99)	15.6 (11.5–21.0)	12.4 (8.4–18.0)	13.7 (9.5–19.5)	–0.0688	0.75
White, non-Hispanic	1.0 (Ref.)	9.0 (6.7–12.0)	8.6 (6.4–11.6)	11.1 (8.0–15.1)	0.1309	0.41
Threatened or injured with a weapon at school (past 12 months)						
Black, non-Hispanic	1.60 (1.07–2.41)	15.6 (8.0–28.1)	15.7 (11.7–20.7)	12.9 (7.2–22.0)	–0.0494	0.89
Hispanic	0.89 (0.63–1.27)	9.0 (5.5–14.3)	9.4 (6.7–13.0)	7.7 (4.9–11.8)	–0.1270	0.61
White, non-Hispanic	1.0 (Ref.)	8.2 (5.5–12.0)	7.1 (4.9–10.1)	12.9 (8.6–18.9)	0.4292	0.09
Bullied at school (past 12 months)						
Black, non-Hispanic	0.31 (0.22–0.44)	21.4 (12.2–34.7)	17.2 (10.6–26.9)	18.2 (12.2–26.2)	–0.0651	0.82
Hispanic	0.56 (0.45–0.70)	31.2 (25.1–38.0)	26.6 (21.0–33.1)	27.6 (23.2–32.5)	–0.1077	0.44
White, non-Hispanic	1.0 (Ref.)	42.2 (34.8–50.0)	40.8 (32.8–49.3)	37.6 (33.6–41.7)	–0.1265	0.31
Electronically bullied (past 12 months)						
Black, non-Hispanic	0.41 (0.31–0.54)	17.0 (12.6–22.6)	16.3 (12.1–21.5)	20.5 (12.5–31.6)	0.1336	0.61
Hispanic	0.55 (0.42–0.71)	24.8 (17.8–33.4)	17.7 (13.3–23.2)	25.4 (20.1–31.5)	0.0619	0.75
White, non-Hispanic	1.0 (Ref.)	36.0 (29.1–43.5)	36.0 (30.1–42.4)	28.2 (22.8–34.1)	–0.2484	0.09
Physical dating violence (past 12 months)						
Black, non-Hispanic	1.19 (0.81–1.75)	14.2 (8.8–22.2)	23.8 (15.5–34.7)	11.6 (7.0–18.6)	–0.1425	0.53
Hispanic	1.13 (0.83–1.54)	22.6 (16.3–30.4)	19.1 (14.2–25.2)	9.8 (5.8–16.2)	–0.7080	0.003
White, non-Hispanic	1.0 (Ref.)	15.3 (11.9–19.5)	14.1 (10.4–18.7)	13.8 (10.6–17.6)	–0.0883	0.55
Sexual dating violence (past 12 months)						
Black, non-Hispanic	0.44 (0.27–0.72)	20.4 (11.5–33.7)	6.4 (3.3–12.0)	10.3 (5.5–18.7)	–0.7987	0.11
Hispanic	0.97 (0.68–1.39)	23.0 (14.8–34.1)	18.6 (11.4–29.0)	18.3 (11.3–28.2)	–0.2334	0.41
White, non-Hispanic	1.0 (Ref.)	22.3 (16.8–29.1)	18.2 (13.7–23.8)	16.7 (11.8–23.1)	–0.2426	0.21
Forced sexual intercourse (lifetime)						
Black, non-Hispanic	0.92 (0.66–1.29)	16.0 (8.3–28.7)	23.9 (17.7–31.6)	15.4 (9.8–23.4)	0.0759	0.77
Hispanic	1.10 (0.85–1.43)	24.0 (18.7–30.3)	21.8 (17.6–26.8)	19.1 (13.3–26.7)	–0.2740	0.17
White, non-Hispanic	1.0 (Ref.)	15.5 (11.5–20.6)	21.0 (16.8–26.1)	21.3 (16.6–26.9)	0.2531	0.12
**Suicide risk behaviors**						
Persistent feelings of sadness or hopelessness (past 12 months)						
Black, non-Hispanic	0.42 (0.33–0.55)	44.8 (35.2–54.7)	52.1 (42.6–61.4)	51.1 (44.6–57.5)	0.1107	0.56
Hispanic	0.69 (0.54–0.89)	58.2 (50.6–65.5)	61.2 (52.9–69.0)	64.1 (56.0–71.4)	0.1766	0.30
White, non-Hispanic	1.0 (Ref.)	67.4 (60.3–73.8)	66.3 (60.9–71.3)	71.6 (65.7–76.8)	0.1679	0.24
Seriously considered attempting suicide (past 12 months)						
Black, non-Hispanic	0.43 (0.34–0.55)	34.4 (25.7–44.3)	28.4 (21.3–36.7)	35.1 (29.2–41.4)	–0.0389	0.85
Hispanic	0.65 (0.54–0.79)	40.7 (34.7–46.9)	45.3 (38.7–52.0)	39.2 (33.2–45.6)	–0.0625	0.63
White, non-Hispanic	1.0 (Ref.)	48.9 (42.2–55.7)	54.1 (50.4–57.6)	52.4 (47.1–57.7)	0.0959	0.44
Made a suicide plan (past 12 months)						
Black, non-Hispanic	0.61 (0.46–0.82)	32.9 (23.6–43.7)	24.1 (16.8–33.2)	36.0 (28.8–43.9)	0.0564	0.81
Hispanic	0.86 (0.69–1.06)	37.9 (31.3–45.0)	35.5 (29.5–42.0)	40.0 (32.8–47.7)	0.0765	0.62
White, non-Hispanic	1.0 (Ref.)	40.1 (34.5–46.0)	42.8 (37.7–48.0)	40.3 (35.8–45.0)	0.0044	0.97
Attempted suicide (past 12 months)						
Black, non-Hispanic	0.97 (0.70–1.34)	29.2 (23.1–36.1)	20.7 (12.5–32.3)	27.2 (18.0–38.8)	0.0293	0.91
Hispanic	1.06 (0.83–1.37)	31.4 (26.4–36.9)	24.6 (18.3–32.2)	23.2 (17.3–30.4)	–0.2610	0.12
White, non-Hispanic	1.0 (Ref.)	28.6 (23.1–34.7)	21.8 (16.4–28.4)	22.3 (18.1–27.3)	–0.2078	0.13
Suicide attempt requiring medical treatment (past 12 months)						
Black, non-Hispanic	0.83 (0.50–1.40)	5.9 (3.2–10.7)	6.4 (3.1–12.9)	7.3 (3.0–16.6)	0.2889	0.50
Hispanic	1.20 (0.83–1.75)	13.3 (9.2–19.0)	8.7 (5.2–14.3)	6.8 (4.3–10.5)	–0.4683	0.05
White, non-Hispanic	1.0 (Ref.)	9.3 (6.4–13.2)	7.5 (5.2–10.8)	5.6 (3.5–8.7)	–0.3545	0.11

**Abbreviations:** aOR = adjusted odds ratio; CI = confidence interval; Ref. = referent group.

* Logistic regression models were used to assess linear trends in the prevalence of violence victimization and indicators of suicide risk among lesbian, gay, and bisexual high school students, by race/ethnicity for 2015–2019, controlling for sex and grade.

^†^ Statistical significance is defined as p<0.05 or a 95% CI that does not include 1.0.

### Suicide Risk

Among all students (Table 2), LGB students had greater odds of suicide risk than heterosexual students across all five indicators, as evidenced by significant main effects for each variable. The percentage of LGB students reporting these outcomes did not vary significantly during 2015–2019.

Among LGB students stratified by sex (Table 3), male students had lower odds of all five suicide risk indicators than female students. Among female LGB students, the percentage reporting suicide attempts decreased significantly from 2015 (32.8%) to 2019 (23.6%). All other trends in suicide risk in these sex-stratified models remained stable.

Among LGB students stratified by race (Table 4), black and Hispanic students had lower odds than white students of reporting persistent feelings of sadness or hopelessness (black, aOR: 0.42; Hispanic, aOR: 0.69) and seriously considering attempting suicide (black, aOR: 0.43; Hispanic, aOR: 0.65). Black LGB students also had lower odds than white LGB students of making a suicide plan (aOR: 0.61). The percentage of LGB students reporting these outcomes in the race/ethnicity-stratified models did not vary significantly during 2015–2019.

## Discussion

Overall, these results underscore that LGB students continue to have a greater prevalence of violence victimization and suicidal behavior than their heterosexual peers. The higher prevalence of violence and suicide among LGB students is consistent with results from other studies regarding sexual minorities and minority stress (*12*,*13*). Minority stress is the preeminent framework for understanding disparities among sexual minorities and refers to the process by which social stigma directed toward LGB and other nonheterosexual persons is enacted through external stressors (e.g., violence, discrimination, or harassment) and internal stressors (e.g., identity concealment or expectations of rejection) (*12*). Both types of stress shape mental and physical health (*12*,*14*), and the impact of violence victimization on LGB youths (*15*) and its connection to elevated suicide risk is well-documented (*16*). LGB students’ disproportionate experience of violence victimization and suicide risk, compared with their heterosexual peers in this study, underscores the continued relevance of minority stress among LGB youths and the continued public health need for action that addresses these sizeable disparities.

Notably, the proportion of LGB students experiencing violence victimization or suicide risk remained fairly stable during 2015–2019. One exception is reports of physical dating violence; fewer LGB students reported experiencing physical dating violence in 2019 than in 2015. This downward trajectory of physical dating violence appears to be a continuation of an already documented population trend of a decrease in experiences of dating violence among adolescents (*17*), and its detection among LGB youths is promising. Regarding suicide risk, a recent study examined local trends since 2009 and reported a decrease in reported suicide risk behaviors among LGB students (*4*). The national trends reported in this analysis warrant continued monitoring over time to assess whether the downward trajectory in local contexts (*4*) reflects the general trajectory of suicide risk and LGB youths nationally.

Results from sex-stratified models highlight important differences between male and female experiences of violence victimization among LGB students. In this sample, male LGB students were more likely to report feeling unsafe at school and being threatened with a weapon; conversely, female LGB students were more likely to report bullying both at school and electronically. This finding is consistent with observational studies of bullying during adolescence; males tend to report more physical forms of bullying and harassment, whereas females tend to report experiencing more verbal and social bullying (*18*). In addition, female LGB students had a greater prevalence of sexual dating violence and forced sex than male LGB students. This sex difference is also consistent with what is known about dating and sexual violence among LGB youths (*7*) and mirrors national trends in dating and sexual violence, in which females are consistently disproportionately affected by these types of victimization (*19*).

Of concern, the percentage of male LGB students who reported being threatened or injured with a weapon at school and who reported forced sexual intercourse significantly increased over time. Although both male and female LGB students are negatively affected by violence, these percentages highlight an increasing trend in violence among male LGB students. Among adults, gay men are at greater risk for physical violence than lesbians (*20*), and the increasing prevalence in these types of violence among male LGB students might suggest an increasing disparity between sexual minority men and women in violence victimization. Continued monitoring of this trend is needed, in both adolescent and adult populations. Female LGB students reported fewer experiences of physical dating violence over time, whereas male LGB students’ reports of experiencing physical dating violence remained stable. This pattern might indicate that the overall reduction in physical dating violence in the population is not occurring among sexual minority males, which might be supported by the data regarding being threatened or injured with a weapon and experiencing forced sex. An assessment of the ways that violence in schools and in dating relationships affects sexual minority males is warranted, both through research to understand underlying mechanisms and in practice to ensure violence prevention programming is directly addressing the needs of sexual minority males.

Despite a trend of decreasing suicide attempts among LGB females during 2015–2019, LGB females consistently reported more suicide risk behaviors than LGB males. This pattern echoes larger population trends in which both adult and youth females report more suicidal ideation than adult and youth males (*21*). Notably, this same literature finds that males experience more deaths by suicide (i.e., completed suicide attempts) than females (*21*); thus, an important remaining question for LGB youths is whether these sex-specific patterns in deaths by suicide hold in this group; however, reliable data regarding sexual orientation and rates of death by suicide are unavailable. Such data could aid in further illuminating how LGB youths are affected by suicide risk behaviors and guide interventions for addressing this public health concern.

In models stratified by race/ethnicity, black and Hispanic LGB students were more likely to feel unsafe and were more likely to be threatened or injured with a weapon than white LGB students. This finding might highlight black and Hispanic LGB students being at greater risk for the forms of victimization that directly compromise physical safety (*18*). Conversely, white LGB students were more likely to report school and electronic bullying, indicating they might be at greater risk for verbal and social victimization. Although the types of racial/ethnic disparities in violence victimization presented in this report do not mirror those reported in previous studies (*9*,*10*), these findings underscore that differences by race/ethnicity among sexual minority youths exist. Schools seeking to address victimization through policies and practices designed to address safety concerns for LGB students can benefit from acknowledging differences in the experiences of LGB youths across races/ethnicities and ensuring all youths are served through these intervention strategies.

Regarding suicide risk, although a significantly lower percentage of black and Hispanic LGB youths reported feeling sad and hopeless or considering suicide than white LGB youths, no differences existed among races/ethnicities in suicide attempts or medically serious suicide attempts. These findings are similar to those from other studies highlighting that all LGB youths are at increased risk for suicide, regardless of race/ethnicity (*10*) and might again highlight the mental health impact of minority stress among all racial/ethnic groups (*12*).

## Limitations

General limitations for the YRBS are available in the overview report of this supplement (*11*). The findings in this report are subject to at least five additional limitations. First, although three cycles of national data to examine trends among LGB youths are available, the brief 2015–2019 period might be inadequate to assess trends. Continued monitoring of these indicators over time to detect progress regarding disparities experienced by LGB high school students is needed. Second, the overall proportion of students identifying as LGB was small: 2015, 8.3% (n = 1,246); 2017, 10.9% (n = 1,494); and 2019, 11.7% (n = 1,531). Therefore, these analyses might be underpowered for detecting statistical differences in trends in models stratified by sex and race/ethnicity. As more data are collected from LGB youths in future cycles of the national YRBS, pooling data across cycles to improve statistical power will be essential for increasing the likelihood of detecting trends in stratified models. Third, this report does not include differences in violence victimization and suicide risk for students who identified their sexual identity as “not sure” or across sexual behavior categories; future studies might benefit from assessing these youths to further understand the experiences of sexual minority students, violence victimization, and suicide risk. Fourth, by pooling 2015–2019 data, the aOR for the difference between groups on all outcomes might mask heterogeneity over time within each subpopulation (e.g., the size of the difference between LGB and heterosexual students might vary between years); however, a disparity between LGB and heterosexual students on these outcomes has been observed since sexual identity data began to be collected on the national YRBS in 2015. Finally, three survey measures had relatively large amounts of missing data in 2019: forced sex (approximately 2,400 observations), sexual dating violence (approximately 3,400 observations), and attempted suicide with injury (approximately 4,900 observations). Most of these missing data can be attributed to some selected schools administering YRBS questionnaire versions that did not include these questions. Consequently, not all students in the national sample were given the opportunity to answer these questions and were counted as missing. 

## Conclusion

These findings highlight the continued need for policies and practices within school environments that reduce victimization and bolster the mental health of LGB students. Substantial evidence exists for the role of antiharassment policies, gay-straight alliances (or other student-led clubs designed to support sexual minority students), and programs aimed at improving staff support of LGB students in improving school environments for these students (*22*). In addition to in-school programs and policies, schools might consider engagement with community organizations and stakeholders to collaborate on implementation of comprehensive violence and suicide prevention strategies that address a range of risk and protective factors at the individual, relationship, community, and societal levels. Comprehensive packages designed to inform these prevention efforts are available from CDC (https://www.cdc.gov/violenceprevention/pub/technical-packages.html). For example, comprehensive approaches to suicide reduction help to prevent suicide risk, support persons at increased risk, prevent reattempts, and help survivors of suicide loss. When refining such practices to meet the needs of LGB students, special consideration of the impact of physical violence on LGB males, suicide risk among LGB females, and the interactions between race/ethnicity and these outcomes is warranted. Furthermore, continued monitoring of these disparities between LGB and heterosexual students over time is needed until these disparities can be eradicated.

## References

[1] Johns MM, Lowry R, Rasberry CN, Violence victimization, substance use, and suicide risk among sexual minority high school students—United States, 2015–2017. MMWR Morb Mortal Wkly Rep 2018;67:1211–5. 10.15585/mmwr.mm6743a4 30383738 PMC6319800

[2] Kann L, Olsen EOM, McManus T, Sexual identity, sex of sexual contacts, and health-related behaviors among students in grades 9–12—United States and selected sites, 2015. MMWR Surveill Summ 2016;65. 10.15585/mmwr.ss6509a1 27513843

[3] Kann L, McManus T, Harris WA, Youth risk behavior surveillance—United States, 2017. MMWR Surveill Summ 2018;67(No. SS-8). 10.15585/mmwr.ss6708a1 29902162 PMC6002027

[4] Raifman J, Charlton BM, Arrington-Sanders R, Sexual orientation and suicide attempt disparities among US adolescents: 2009–2017. Pediatrics 2020;145:e20191658. 10.1542/peds.2019-1658 32041815 PMC7049939

[5] Demissie Z, Rasberry CN, Steiner RJ, Brener N, McManus T. Trends in secondary schools’ practices to support lesbian, gay, bisexual, transgender, and questioning students, 2008–2014. Am J Public Health 2018;108:557–64. 10.2105/AJPH.2017.304296 29470123 PMC5844405

[6] CDC. School Health Profiles 2018: characteristics of health programs among secondary schools. Atlanta, GA: US Department of Health and Human Services, CDC; 2019. https://www.cdc.gov/healthyyouth/data/profiles/pdf/2018/CDC-Profiles-2018.pdf

[7] Dank M, Lachman P, Zweig JM, Yahner J. Dating violence experiences of lesbian, gay, bisexual, and transgender youth. J Youth Adolesc 2014;43:846–57. 10.1007/s10964-013-9975-8 23861097

[8] Walters ML, Chen J, Breiding MJ. The National Intimate Partner and Sexual Violence Survey (NISVS): 2010 findings on victimization by sexual orientation. Atlanta, GA: US Department of Health and Human Services, CDC, National Center for Injury Prevention and Control; 2013. https://www.cdc.gov/violenceprevention/pdf/NISVS_Report2010-a.pdf

[9] Whitton SW, Newcomb ME, Messinger AM, Byck G, Mustanski B. A longitudinal study of IPV victimization among sexual minority youth. J Interpers Violence 2016;34:912–45. 10.1177/0886260516646093 27147275 PMC6538483

[10] Mueller AS, James W, Abrutyn S, Levin ML. Suicide ideation and bullying among US adolescents: examining the intersections of sexual orientation, gender, and race/ethnicity. Am J Public Health 2015;105:980–5. 10.2105/AJPH.2014.302391 25790421 PMC4386523

[11] Underwood JM, Brener N, Thornton J, Overview and methods for the Youth Risk Behavior Surveillance System—United States, 2019. In: Youth Risk Behavior Surveillance—United States, 2019. MMWR Suppl 2020;69(No. Suppl 1). 10.15585/mmwr.su6901a1 PMC7440204 32817611

[12] Meyer IH, Frost DM. Minority stress and the health of sexual minorities. In: Patterson CJ, D’Augelli AR, eds. Handbook of psychology and sexual orientation. New York, NY: Oxford University Press; 2013: 252–66.

[13] Bouris A, Everett BG, Heath RD, Elsaesser CE, Neilands TB. Effects of victimization and violence on suicidal ideation and behaviors among sexual minority and heterosexual adolescents. LGBT Health 2016;3:153–61. 10.1089/lgbt.2015.0037 26789401 PMC4841901

[14] Frost DM, Lehavot K, Meyer IH. Minority stress and physical health among sexual minority individuals. J Behav Med 2015;38:1–8. 10.1007/s10865-013-9523-8 23864353 PMC3895416

[15] Zaza S, Kann L, Barrios LC. Lesbian, gay, and bisexual adolescents: population estimate and prevalence of health behaviors. JAMA 2016;316:2355–6. 10.1001/jama.2016.11683 27532437 PMC5541373

[16] Burton CM, Marshal MP, Chisolm DJ, Sucato GS, Friedman MS. Sexual minority-related victimization as a mediator of mental health disparities in sexual minority youth: a longitudinal analysis. J Youth Adolesc 2013;42:394–402. 10.1007/s10964-012-9901-5 23292751 PMC3570607

[17] CDC. Youth Risk Behavior Survey: data summary & trends report 2007–2017. Atlanta, GA: US Department of Health and Human Services, CDC, Division of Adolescent and School Health; 2018. https://www.cdc.gov/healthyyouth/data/yrbs/pdf/trendsreport.pdf

[18] Carbone-Lopez K, Esbensen F-A, Brick BT. Correlates and consequences of peer victimization: gender differences in direct and indirect forms of bullying. Youth Violence Juv Justice 2010;8:332–50. 10.1177/1541204010362954

[19] Smith SG, Zhang X, Basile KC, The National Intimate Partner and Sexual Violence Survey (NISVS): 2015 data brief—updated release. Atlanta, GA: National Center for Injury Prevention and Control, CDC; 2018. https://www.cdc.gov/violenceprevention/pdf/2015data-brief508.pdf

[20] Stotzer RL. Comparison of hate crime rates across protected and unprotected groups—an update. Los Angeles, CA: University of California at Los Angeles, The Williams Institute; 2012. https://escholarship.org/uc/item/43z1q49r

[21] Beautrais AL. Gender issues in youth suicidal behaviour. Emerg Med (Fremantle) 2002;14:35–42. 10.1046/j.1442-2026.2002.00283.x 11993833

[22] Johns MM, Poteat VP, Horn SS, Kosciw J. Strengthening our schools to promote resilience and health among LGBTQ youth: emerging evidence and research priorities from The State of LGBTQ Youth Health and Wellbeing Symposium. LGBT Health 2019;6:146–55. 10.1089/lgbt.2018.0109 30958731 PMC6551982

